# Disgust assessment: Factorial structure and psychometric properties of the French version of the Disgust Propension and Sensibility Scale Revised-12

**DOI:** 10.1371/journal.pone.0210639

**Published:** 2019-01-28

**Authors:** Caroline Novara, Julie Boiché, Cindy Lebrun, Alexandra Macgregor, Yohan Mateo, Stéphane Raffard

**Affiliations:** 1 Univ Paul Valéry Montpellier 3, Univ. Montpellier, EPSYLON EA, Montpellier, France; 2 Groupe Ramsay Gds, Clinique RECH, Montpellier, France; 3 Service Universitaire de Psychiatrie Adulte, CHU Montpellier, Montpellier, France; University of Florence, ITALY

## Abstract

The present study examined the internal and external validity of the French version of the 12-item Disgust Propensity and Sensitivity Scale-Revised (DPSS-12) in a nonclinical sample from the general population. Two hundred and eighty-two participants completed the DPSSf-12 questionnaire as well as the Anxiety Sensitivity Index (ASI), Anxiety Trait (STAI B), Obsessional Belief Questionnaire 44 items (OBQ 44), Obsessive Compulsive Inventory-Revised (OCI-R) and Positive and Negative Affect Schedule (PANAS). Confirmatory Factor Analysis supported a 2-factor structure after two sensitivity items were removed. The 10-item scale showed good internal consistency, construct validity and test-retest reliability. These adequate psychometric properties make the DPSSf-10 appropriate for use by researchers and practitioners.

## Introduction

Disgust is recognized as a universal emotion [1, 2], with distinct developmental features, behavioral, physiological dimensions and cognitive biases [3,4,5,6,7,8]. The theoretical model put forward by Rozin, Haidt, and McCauley [9]represents the main reference which has inspired literature on disgust over the last decades. From an evolutionary perspective, there is a broad consensus that disgust plays a key role in motivating behavior that reduces exposure to pathogens, and this concept has developed as a mediator of a dynamic adaptive system, a “behavioral immune system”, motivating disease avoidance [10, 11, 12, 13].

From a clinical perspective, disgust has been shown to be involved in the development and maintenance of several mental disorders including spider phobia [14, 15], contamination-based Obsessive–Compulsive Disorder (OCD; [16, 17], Blood-Injury-Injection (BII) phobia [18], hypochondriasis [19], Post Traumatic Stress Disorder [20], sexual dysfunctions disorders [21] and eating disorders [22].

Despite the fact that disgust represents a public health concern [11], very few studies have examined disgust in certain countries, such as France. For example, it seems that emotional reactions linked to disgust played a major role regarding the consumption of beef during The Mad Cow disease crisis in France [23]. One of the reasons why French researchers and clinical practitioners have paid little attention to disgust is the lack of a formal measurement tool to assess individual’s tendencies to disgust reactions. Such a tool would enable examining the role of disgust in some disgust-relevant psychopathological conditions, thus enabling to verify the applicability of scientific knowledge concerning the role of disgust in psychopathology as it was demonstrated in other cultural spheres. The construction of a standardized measure instrument for the French general population is thus necessary, because it will not only enable investigations of the specific characteristics of disgust in France, but also provide elements upon shared cross- cultural components of disgust.

It is now well admitted that disgust can be distinguished between Disgust Sensitivity (DS, i.e., the extent to which an individual is embarrassed to feel disgust) and Disgust Propensity (DP, i.e., trait disgust or the tendency to experience disgust frequently and intensely) [24, 25]. The Disgust Propensity and Sensitivity Scale (DPSS) has been specifically developed in English to assess both DS and DP and has been validated in various languages such as Japanese [26], Dutch [25], or Italian [27].

The original DPSS scale consisted of 32 items used to measure DS and DP with 16 items per factor. The scale demonstrated good psychometric properties, including a good internal consistency for both the total scale and its subscales [28]. Later factor analyses, however, conducted to the proposition of two abbreviated versions. First, Van overveld, De Jong, Peters, Cavanagh and Davey [25] proposed a 16-item scale (DPSS-R) with two subscales of 8 items for DS and DP factors. The item selection process was not only data driven, but also lead by theoretical considerations. Olatunji, Cisler, Deacon, Conolly, and Lohr [29] conducted exploratory analyses and found that 4 items loaded on a different factor, compared to the results obtained by Van overveld et al. [25]. Fergus and Valentiner [24] reported similar results, and suggested that a 12-item version, without these 4 items, guaranteed better psychometric properties. Taken together, the evidence provided by previous psychometric properties suggest that DPSS-12 is the most valid measure to assess sensitivity and propensity to disgust among available versions [30].

Recently, Goetz, Cougle, and Lee [31] suggested that heterogeneous items included in the DPSS-12 question the adequacy of the scale. Indeed, although the DPSS-12 did reveal a DS and a DP factor, two items did not load on them. The authors pointed out that the DPSS-10 produces a better consistency than a one-dimension version, or a two-or three -factor version with the 12 items. Altogether, recent work on the DPSS-12 supported the presence of 2 distinct factors, DS and DP, for which good reliability was showed. However, the loading of all items within this, 12-item version appears susceptible to cultural specificities. Indeed, although basic emotions are expected to be similar among all individuals, certain emotional components are likely to be influenced by the environment and the individual's experiences [32]. It has been suggested that culture may influence the type of stimulus triggers, display codes, associated affects, and behavioral consequences [33]. Although general themes of disgust triggers seem to be consistent across cultures, some variations appear in the subjective experience of disgust emotion as well as in specific triggers [34, 35]. The peculiarity of cultural specificities in regard of the emotion of disgust has furthermore been advanced as a valid argument by Fergus and Valentiner [24] who assumed that the factorial differences observed in Dutch and American populations could partly be explained by cultural specificities. This underlines the importance of examining this issue specifically in the French population.

Significant associations with phobias related to disgust, and especially with contamination concerns and other relevant psychopathological traits (sensitivity to anxiety), have been enlighten and allowed then to study the unity of these constructs under this framework. However, the heterogeneity of the psychometric data obtained suggests a need for clarification. Validating a French version of the DPSS-12 would bring new information on the validity of the analyzed constructs, their applicability and their generalization to a French general population. In order to assess the external validity of the scale, we chose to focus on obsessive-compulsive symptomatology in its behavioral and cognitive dimensions, as well as on anxiety. The cognitive models of OCD have been suggested to provide a useful starting point for the examination of potential thought processes in pathological disgust responses. These models posit that the vast majority of the population has intrusive, undesirable thoughts that are similar to the content of obsessions experienced by people with OCD [36]. It is therefore not the content of thoughts but the interpretation of the personal meaning of thoughts, or their responsibility for the perceived consequences causing distress and repetition, that characterize OCD [37]. Beliefs about intrusive obsessive thoughts have therefore been suggested as a natural parallel to interpretations of disgust reactions [38].

In previous studies, it has been suggested that high disgust propensity would play a specific role in OCD [29]. High DP was found to be significantly higher in OCD linked to contamination (C-OCD) than in other subtypes of OCD [39]. However, many studies that have investigated this issue only evaluated DP and did not include a measure of DS in their assessments [40, 41]. Recent research has suggested that DS may also be associated with C-OCD symptomatology and is, in addition, associated with a general inability to regulate one's emotions [42]. DP has been found to be uniquely associated with OCD when compared with general anxiety disorder, indicating that DP may be more specific to OCD than to generalized anxiety [43]. Moreover, a strong DS was correlated with a high sensitivity to anxiety, present in anxiety disorders, suggesting that the DS could be more general in anxiety disorder and less specific to C-OCD.

The objective of this study was to examine the psychometric properties of the French version of the DPSS-12. First, we examined factorial structure using Confirmatory Factor Analysis. More precisely, we tested the fit of a 2-factor model (i.e., DS and DP) containing 6 items each [24] in a large sample of French participants from the general population.

Next, we evaluated external validity and more precisely we hypothesized that DP would be more strongly associated with behavioral dimensions of OCD symptomatology as measured by OCI-R than DS, and that DS would be more strongly associated with cognitive dimensions of OCD symptomatology as assessed by OBQ-44 and cognitive sensitivity related to anxiety (ASI) than DP. Although previous studies suggest a strong association of C-OCD with anxiety [44], our assumption is that the relationship between washing compulsion and disgust will be maintained while controlling for anxiety.

Last, in order to examine the temporal reliability of the scale, we assessed disgust twice with a 2-month interval in the participants, using the French DPSS-12. Considering that disgust is conceived as a relatively stable individual disposition, we assumed that the scores would be stable over this short time period.

## Material and method

### Participants

Two hundred and eighty-four adults voluntarily completed self-reported questionnaires containing the 12 items of the DPSSR as the main measure of interest. All participants provided written informed consent, prior to the experiment. The research has been submitted to the members of the GCS Ramsay Générale de Santé for Education and Research Scientific Orientation Committee and has received a favorable opinion on 18/10/18. The committee testifies that the study appears to be in accordance with the Scientifics principles generally accepted and to the ethical standards of research. IRB’s number which was assigned is: COS-RGDS-2018-10-002-Avis IRB-NOVARA-C. We relied both on a secured online research platform set up by the Epsylon laboratory and paper questionnaires filled in small groups to recruit participants from the general population (187 females, 72 males, 25 did not provide gender, *M*age = 31.39; *SD*age = 13.38 ranging from 19 to 77; 23,1% of whom had a level of education lower than bachelor's degree and 62% of whom were undergraduate students).

#### Measures and procedure

In order to adapt the scale in French, a standard procedure of translation and back-translation was carried out. At first, two expert psychologists independently translated the scale from English to French. The different translations were compared to obtain a first consensual translation of the tool. A bilingual expert then translated the French version of the questionnaire into English. Finally, the back-translation was submitted and validated by Dr Van Overveld. In order to examine the stability of the scale over time, the DPSSf was administered twice two months apart. In addition, participants completed the following scales:

**Anxiety sensitivity Index Revised (ASI-R)** [45], was used to measure individual differences in sensitivity to anxiety. A 2-factor structure was observed for the French version of this scale [46]: "Fear of the consequences related to the physical sensations of Anxiety"(CP) includes 19 items, and "Fear of Social and Cognitive Consequences of Anxiety"(CSC) includes 17 items rated on a 5-point likert scale (from “very little” to “very much”). In this study, the internal consistency of both subscales (CP subscale, Cronbach’s α = .87; CSC subscale, Cronbach’s α = .93) was adequate.

**State-Trait anxiety inventory (STAI-B)** [47] is a widely used inventory for assessing individual differences in trait anxiety, containing 20 items rated on a 4-point Likert scale (from 1 "Not at all" to 4 "Extremely"). The internal consistency was satisfactory in the current sample (Cronbach’s α = .89).

**Obsessional Belief Questionnaire 44 (OBQ-44)** [48] The OBQ-44 is a self-reported 44-item questionnaire used to assess the presence and severity of obsessive beliefs associated with OCD rated on a 7-point likert scale (from “strongly disagree” to “strongly agree”). Three subscales assess the beliefs associated with the over-importance of the need to control one's thoughts (control of thought), the beliefs about an increased sense of responsibility to prevent danger or perceived threats (responsibility), and the beliefs about the need for perfectionism and intolerance to uncertainty (perfectionism). In this sample, the internal consistency was satisfactory (respectively, Cronbach’s α = .89, .89 and .88) for all subscales.

**Obsessive-compulsive Inventory-Revised (OCI-R)** [49] is an 18-item self-reported scale used to measure the severity of obsessive symptoms, through six 3-item subscales: washing, checking, obsessive thoughts, neutralization, hoarding and symmetry, rated on a 5-point likert scale (from “not at all” to “extremely”). The internal consistency in this study ranged from .59 to .82 (*M* = .73)

**Positive affects and negative affects Schedule (PANAS)** [50] is a self-reported scale containing 20 items measuring affective states. This scale contains two subscales assessing respectively positive affects and negative affects. The PANAS items are scored on a 5-point Likert scale (ranging from 1 "Not at all or very little" to 5 "Extremely"). The internal consistency of the two subscales was satisfactory (PANAS-positive affect, Cronbach’s α = .82; PANAS-negative affect, Cronbach’s α = .87)

### Data analysis

First, a series of preliminary analysis were run. We examined missing data and checked the presence of univariate and multivariate outliers. The normality of the distribution was verified through the distribution kurtosis and skewness for each item. Potential collinearity was tested through Pearson correlations. Next, using R software [51],a Confirmatory Factorial Analysis (CFA) for continuous data using a maximum likelihood estimation method with robust (Huber-White) standard errors and a scaled test statistic that is (asymptotically) equal to the Yuan-Bentler test statistic was applied to examine if a 2-factor model showed adequate fit with the observed data. We estimated the model of interest through the commonly used indices, following Kline [52]: the Akaike information criterion (AIC), the comparative fit index (CFI,), the Tucker-Lewis index (TLI), the root mean square error approximation (RMSEA) RMSEA value was supplemented with a confidence interval (CI). In presence of good fit, the suggested lower limit of the RMSEA’s CI falls at or below .05 and upper limit should not exceed .10.

There is no definitive cut-off point for these indices although recommended thresholds are: for AIC (the model with the weaker score show the best parsimony principle), CFI> .90, TLI> .90, RMSEA< .05. We further submitted the different models to the ratio Chi square on freedom degree (χ^2^/df< 3.00).

Second, to test external validity, using SPSS, we examined the correlations between the DPSSf factors and other measures of anxiety, and obsessive-compulsive symptomatology through Pearson’s correlations. We then tested the significance of differences between correlation coefficients to shed light on the utility of the factorial solution proposed as presenting relevant dimensions subscales of DPSSf through William’s test. As the behavior commonly associated with C-OCD is compulsive washing, we used regressions to measure the predictive value of DS and DP on washing dimension of the OCI-R, controlling for the scores obtained regarding anxiety sensitivity (ASI-R CP, ASI-R CSC), trait anxiety (STAI B), and cognitive distortions relevant for OCD (OBQ 44: perfectionism, responsibility and control of thoughts).

Last, in order to examine the temporal reliability of the scale we examined Intra-Class Coefficients, considering values >.75 as satisfactory [53].

## Results

### Preliminary analyses

For the preliminary analysis of the data we used recommended criteria from Tabachnik and Fidell [54]. Two participants were excluded because they had more than 10% missing data, Multiple imputations, using the SPSS Impute Missing Data Values module, were operated for 10 participants who had less than 10% missing data. Examination of standardized scores revealed the presence of 14 simple outliers (standardized score ± 3.29). Their score were replaced by the corresponding extreme, within a range of normal value for the identified items. No response pattern indicated the presence of multiple outliers were (distance Mahalanobis significant at p < .001). The main analyses were launched on a final sample of 282 participants. Skewness values were ranged from -.707 to 1.798, and kurtosis values were comprised between -1.015 to 2.192, indicating a deviation from normality. Therefore, a Confirmatory Factor analysis using a maximum likehood robust method was considered appropriate. Because all correlations were below .85, it was concluded that there was no multicollinearity in the dataset.

### Confirmatory factor analysis

A two-factor model was tested (Model 1), including the estimation of the 12 target loadings, 2 factors variance, correlations between the 2 factors, as well as uniqueness values for all 12 items. For identification purpose, the loading between the first indicator of each latent construct and its target factor were fixed at 1.0. This model displayed only partly convincing fit Robust χ^2^(53) = 105, χ^2^ / df = 1.98; AIC = 8334.3, Robust CFI = .91, Robust TLI = .89, Robust RMSEA = .06, 90% Robust CI RMSEA = [.04-.06]. An examination of the factor loadings enabled to identify one item that that did not show salient loading on the expected factor (see Table 1). Even if only item 12 was below this threshold in the 2*6 factor model, when running a 11-item model, it appeared that (1) some indicators of fit were not entirely satisfactory (significant robust χ^2^) and (2) the factor loading of item 11 did not reach the cut-off anymore.

**Table 1:** Confirmatory factor analysis and item statistics of the disgust propensity and sensitivity scale revised-12 (DPSS-12) (*N* = 282).

Items description	M1	M2
***Disgust Propensity***		
1. J’évite les choses dégoutantes.	**.58**	**.58**
*I avoid disgusting things*.		
4. Je ressens de la répulsion.	**.67**	**.67**
*I feel repulsed*.		
5. Les choses dégoutantes me retournent l’estomac.	**.64**	**.64**
*Disgusting things make my stomach turn*.		
6. Je grimace quand je ressens du dégoût.	**.53**	**.53**
*I screw up my face in disgust*.		
8. Je ressens du dégoût	**.70**	**.70**
*I experience disgust*.		
10. Il y a des choses que je trouve dégoutantes.	**.53**	**.53**
*I find something disgusting*.		
***Disgust Sensibility***		
2. Quand je me sens dégouté, j’ai peur de m’évanouir.	**.40**	**.46**
*When I feel disgusted*, *I worry that I might pass out*.		
3. J’ai peur quand j’ai la nausée.	**.63**	**.68**
*It scares me when I feel nauseous*.		
7. Quand je m’aperçois que j’ai la nausée, j’ai peur de vomir.	**.65**	**.72**
*When I notice that I feel nauseous*, *I worry about vomiting*		
9. Cela m’effraie de me sentir faiblir	**.50**	**.46**
*It scares me when I feel faint*.		
11. Je me sens gêné(e) lorsque je me sens dégouté(e)	.34	_
*It embarrasses me when I feel disgusted*.		
12. Je pense que ressentir du dégoût est mauvais pour moi	.25	_
*I think feeling disgust is bad for me*.		
**α Propension**	.77	.77
**α Sensibility**	.57	.63

M1 = Model 1, DPSS-12, Fergus & Valentiner, 2009; M2 = Model 2, DPSSf-10

A two factor model (Model 2) including 6 items on the first factor (DP) and 4 items on the second (DS) was thus tested, leading to satisfactory fit indices: Robust χ^2^ = 46.67, df = 34, χ^2^ / df = 1.40; AIC = 8812.72, Robust CFI = .97, Robust TLI = .96, Robust RMSEA = .03, Robust CI RSMSEA = [.00-.04]. All 10 items demonstrated distinctive and salient loadings ranging from .46 to .72, with an average of .60 onto one of the two factors (see Table 1). The interfactor correlation between DS and DP was significant (*r* = .39). Cronbach’s alpha values for the two subscales (α _DP_ = .77; α _DS_ = .63) and for the total score (*α* = .76) were acceptable.

### External validity

The correlations between disgust scores and measures of anxiety (ISA, STAI) and obsessional-compulsive symptomatology (OCI-R, OBQ 44) are shown in Table 2. The total DPSSf-10 score was significantly related to anxiety levels, behavioral symptoms and cognitive distortions of OCD. If the DP factor was strongly correlated with washing and symmetry dimensions of the OCI-R, the DS factor was strongly linked with the anxiety measures and the cognitive distortions of OCD.

**Table 2:** DPSSf-10 correlations with convergent and discriminant measures.

SCALES		DP^a^	DS^a^	TOTAL SCORE^a^	t value^b^
STAI B		.26**	.30**	.34**	-0.64
ASI					
	CS	.34**	.50**	.49**	-2.80**
	CPC	.29**	.46**	.43**	-2.89**
OBQ					
	Responsability	.08	.22**	.17**	-2.17*
	Control of thoughts	.20**	.07	.14*	2
	Perfectionnism	.16**	.21*	.24**	-0.77
OCI-R					
	Checking	.10	.05	.09	0.76
	Washing	.25**	.16*	.25**	1.40
	Thought	.18**	.23**	.24**	-0.78
	Neutralization	.04	.11	.09	-1.06
	Symetry	.23**	.15*	.23**	1.24
	Hoarding	.03	.11	.08	-1.22
PANAS					
	Positive Affects	-.14*	-.04	-.11	-1.53
	Negative Affects	.18**	.29**	.27**	-1.88*

STAI B = State trait anxiety inventory; ASI CS = inventory sensitivity to anxiety fear of the social consequences; ASI CPC = inventory sensitivity to anxiety fear of the of cognitive and physical consequences; OBQ = obsessive beliefs questionnaire; OCI = obsessive compulsive inventory; PANAS = positive and negative affects schedule; DP = Disgust Propension; DS = Disgust sensibility. (N = 282)

^a^ Pearson’s correlation

^b^ William’s test for correlation comparison

** p < .001

*p < .05

A regression analysis was performed, entering simultaneously as predictor variables DS and DP scores, as well as negative affects, cognitive distortions of OCD (perfectionism, responsibility and control of thoughts), and anxiety trait scores. Together, the seven predictor variables explained a significant proportion of the variance in washing (*r*^*2*^ = .20, *F*(8,256) = 7.93, *p* < .001) assessed with the OCI. After controlling for the other variables, the amount of unique variance of DP as a predictor for OCI washing score is statistically significant (*β* = .19, *t* = 3.34, *p* = 0.001), in a model that also significantly include perfectionism and intolerance to uncertainty.

### Temporal reliability

The ICC computed between the DPSS total scores (*r* = .77 CI [.61 - .86]; *p* < .001) and the two subscales scores (DP, *r* = .74 CI [.62 - .82]; *p* < .001; DS, *r* = .76 CI [.65 - .84]; *p* < .001) were significant and high, suggesting adequate temporal stability.

## Discussion

The aim of this study was to examine the psychometric properties of the French version of the DPSS-12 in a sample of participants from the general population. After the removal of two items, two latent constructs emerged from our analyses: (a) disgust propensity (DP), (b) disgust sensitivity (DS). This 10-item factorial solution produced by Confirmatory Factor Analysis provides a correctly fitted model across multiple indexes when compared to the 12-item model of the English version.

### Factor structure

Contrary to previous factor analyses offering a 12-item scale [24], items 11 and 12 appeared problematic. However, as previously observed by Goetz et al. [31], if items 11 et 12 do not function in the way they were originally intended by Fergus and Valentiner [24], a plausible explanation may be that they are less conceptually related to their purported factors compared to other items, and might be related to another disgust related dimension.

The DP dimension (items 1, 4, 5, 6, 8, 10) was stable as it has been previously shown in the different validations of this scale [24, 31, 30], and was correctly associated with the tendency to have overactivated reactions of disgust toward various stimuli. The DS dimension (items 2, 3, 7, 9) corresponds, in the French validation of the scale, to items that are specifically sensitive to autonomic activation, such as fear or nausea, linked to feelings of disgust (« *It scares me when I feel nauseous*.»).

### Reliability and external validity

We examined the reliability of the sub-dimensions of the DPSSf-10 to estimate Cronbach’s alpha coefficient as parameter of internal consistency, and intra-class correlation coefficient as the parameter of test-retest reliability. The results are satisfactory and represent a substantial improvement on the original DPSS-12. To test the convergent and discriminant validity of the DPSSf-10, correlations and regression analyzes were performed and showed that the two concepts, although correlated, have specific associations with distinct constructs. As hypothesized, and consistently with previous work [29], DP was more strongly associated with the behavioral dimension of OCD symptomatology. Also, the propensity factor was correlated with washing compulsion even when anxiety, cognitive distortions and negative affects were controlled. DS was more strongly associated with the cognitive dimensions of OCD symptomatology (responsibility and perfectionism), with anxiety trait, and with both physical concern and social and cognitive concerns of anxiety sensitivity, suggesting that DP may be more specific to C-OCD, whereas DS may be more generally associated with anxiety disorders. Finally, the DPSSf-10 showed no association with positive affects.

These results provide valid support for the construct validity of the DPSSf-10 and the conceptual independence of its subscales. The consistency in findings across studies that the DP and DS scales are uniquely related to different constructs firmly supports the notion that DP and DS possess differential predictive value. Overall, these findings suggest that the DP and DS scales adequately assess disgust per se and do not appear redundant with fear-based constructs as assessed by anxiety trait or anxiety sensitivity scales. The use of this scale therefore opens up the possibility for researchers to examine the similarities and differences in the spectrum of anxiety disorders with particular attention to the behavioral characteristics and somatic disgust sensitivity.

### Limitations

First, as mentioned above, it has been suggested that the heterogeneity of the results in characterizing the experience of disgust could be attributed to cross-cultural differences [55, 56]. Thus, the proposed DPSSf-10 in this article might reflect a specific cultural trait in the treatment, recognition, conceptualization and verbalization of disgust. In fact, its validity is limited to the French general population. However, the DPSSf-10 may be useful for future research in clinical populations or other groups of healthy subjects to assess the invariance of scale measures. Furthermore, our sample was relatively young and included a large majority of females. A study involving a more representative sample of the French population, including more males and elderly people, would further help understand the experiences of disgust sensitivity and disgust propensity in these populations. Finally, the presence of common method variance (i.e., self-report) may have inflated the correlations among the study variables.

#### Perspectives

Berle, Phillips [57], Olatunji [58, 59] suggested that a complete emotional assessment must take into account cognitive (threat interpretation), behavioral (safety behavior) and physiological (nervous parasympathetic system activation patterns) dimensions to adequately explore the disgust phenomenon. Yet, the French validation of the scale excluded two items representative of cognitive sensibility (items 11 and 12). In the previous validation of DPSS-12 [31] the original sensitivity factor was decomposed into two sub-dimensions including somatic sensitivity and ruminative / self-disgust, comprising these two items, describing more specific cognitive sensibility. This factorial solution, although theoretically justified, seems nevertheless to be statistically fragile. A refinement of the scale in line with these recommendations, by inserting items of a nature to reflect the cognitive assessments that individuals make of an experience of disgust, could thus be relevant.

### Conclusion

Disgust is a complex emotion and plays an important role in the development and maintenance of various psychopathological conditions and specifically C-OCD. The development of adequate tools providing a dimensional assessment of the forms of disgust regardless of their context of appearance is of importance not only to increase the volume of research on this subject but also to improve the management of this emotion that patients as well as practitioners are not used to identify. Provide a standardized index of the magnitude of the emotional experience regardless of its onset context, taking into account its cognitive, behavioral, and physiological dimensions, is an important effort that research must carry out. Evaluation tools should continue to be optimized to provide valuable data to guide and adjust clinical interventions.

## Supporting information

S1 Data Complete raw data can be found on COMPLETE DATA DPSSf. (XLSX)
